# Salvage Treatment for Extragonadal Germ Cell Tumours: High-Dose Chemotherapy and Autologous Stem Cell Transplantation Outcomes—A Single-Centre Experience

**DOI:** 10.3390/jcm13216494

**Published:** 2024-10-29

**Authors:** Alper Topal, Ismail Erturk, Caglar Koseoglu, Aysegul Dumludag, Ömer Faruk Kuzu, Berkan Karadurmus, Esmanur Kaplan Tuzun, Huseyin Atacan, Nurlan Mammadzada, Gizem Yildirim, Ramazan Acar, Nuri Karadurmus

**Affiliations:** Division of Medical Oncology, Department of Internal Medicine, Gulhane Research & Training Hospital, Ankara 06010, Türkiye; ierturk@hotmail.com (I.E.); drcaglarkoseoglu@hotmail.com (C.K.); dr.aysegulcomakli@gmail.com (A.D.); drfarukkuzu@gmail.com (Ö.F.K.); berkankaradurmus@gmail.com (B.K.); esmanurkaplan@hotmail.com (E.K.T.); drhuseyinatacan@gmail.com (H.A.); mammadzadanurlan@gmail.com (N.M.); gizemyildirim_@hotmail.com (G.Y.); ramazanacar@hotmail.com (R.A.); drnkaradurmus@yahoo.com (N.K.)

**Keywords:** high-dose chemotherapy, germ cell tumours, stem cell transplantation, salvage therapy

## Abstract

**Objective:** Extragonadal germ cell tumours have a more unfavourable prognosis than gonadal germ cell tumours. We aimed to evaluate the survival analysis, response rates, and factors affecting responses to high-dose chemotherapy (HDCT) and autologous stem cell transplantation (ASCT) in patients with relapsed/refractory extragonadal germ cell tumours. **Methods:** This study included patients diagnosed with extragonadal germ cell tumours who underwent HDCT + ASCT between November 2016 and January 2023 at Gülhane Training and Research Hospital. Clinical characteristics and follow-up data from patient records and the hospital’s electronic system were retrospectively analysed. Patients under 18 years of age and those without medical records were excluded. Patient characteristics, post-HDCT progression-free survival (PFS), overall survival (OS) data, and factors affecting survival were examined. The relationship between clinical factors and OS/PFS was analysed. **Results:** Twenty-five patients were included in this study. Complete response (CR) was observed in seven patients (28%), partial response (PR) was observed in nine patients (36%), stable disease (SD) was observed in one patient, and progressive disease (PD) was observed in eight patients (32%) after HDCT + ASCT. The median follow-up period was 25.4 months. The median PFS and OS after HDCT + ASCT were calculated as 6.1 months and 12.2 months, respectively. **Conclusions:** Salvage HDCT + ASCT is an option in the treatment of extragonadal germ cell tumours, offering the potential for prolonged survival and curing.

## 1. Introduction

Testicular cancer is the most common solid malignancy in men aged 15–35 years, and 95% of this cancer presents in the form of germ cell tumours (GCTs) [1,2]. Male GCTs generally originate in the testis. However, 2% to 5% of them are of extragonadal origin [3]. The location of extragonadal germ cell tumours (EGGCTs) varies according to age. In adults, they tend to occur in the midline of the anterior mediastinum, the retroperitoneal region, and the suprasellar and pineal regions [4,5]. Like gonadal germ cell tumours, EGGCTs occur in histological types similar to GCTs. These include seminomatous (germinoma/dysgerminoma) and nonseminomatous germ cell tumours, including endodermal sinus tumours, yolk sac tumours, embryonal carcinomas, choriocarcinomas, and mature or immature teratomas [6]. Testicular tumours and EGGCTs also share similar serological features, such as secretion of the tumour markers alpha-fetoprotein (AFP) and beta-human chorionic gonadotropin (β-HCG) [7].

Although EGGCTs have similar histological, serological, and cytogenetic features to those of gonadal GCTs, their clinical characteristics, behaviour, and biology are different. EGGCTs have worse chemosensitivity and prognosis than gonadal tumours, especially nonseminomatous tumours [8,9]. Primary mediastinal and retroperitoneal seminomatous EGGCTs have an equivalent prognosis to their primary gonadal counterparts. However, nonseminomatous EGGCTs have a worse prognosis than that of seminomatous EGGCTs [7].

Treatment of EGGCTs is similar to that of gonadal GCTs. After histological classification into seminoma and non-seminoma, chemotherapy (CT) is administered according to the risk classification. Surgical resection may also be performed in patients with a residual tumour after treatment. Efficacy and survival increase with multimodal treatment options [10,11]. There is no standard salvage treatment for recurrent/refractory EGGCT patients. Second-line treatment options include high-dose chemotherapy and autologous stem cell transplantation (HDCT and ASCT) or conventional-dose chemotherapy (CDCT) regimens containing ifosfamide plus cisplatin and another agent such as paclitaxel, etoposide, or vinblastine [12,13,14]. The most effective regimen as a second-line therapy for relapsed/refractory patients is still controversial [14]. One of the key challenges in the field of salvage therapy is identifying which patients would benefit most from treatment with CDCT or HDCT. Despite the growing preference for high-dose chemotherapy and autologous stem cell transplantation over the last three decades, the optimal timing and number of HDCT (single vs. double or triple sequential approach) remain a topic of contention, with significant variations in practice [15]. Given the rarity of this type of cancer and the low rates of recurrence or resistant disease, the results of subsequent treatment steps are limited by the small number of patients. The retrospective nature of the data makes it challenging to compare treatment strategies, and thus, extrapolation is further limited [16]. Clinicians typically base their decisions on the results of non-randomised studies, the experience of the centre, and the prognostic factors of the patient. HDCT and ASCT are generally preferred for cases with poor prognostic factors. The rationale for utilising HDCT in conjunction with salvage ASCT is predicated on the understanding that GCTs exhibit sensitivity to chemotherapeutic agents. However, high-dose chemotherapy and total body irradiation eventually result in dose-limiting haematopoietic toxicity. The tolerable dose of chemotherapy can be increased by the use of ASCT rescue. Therefore, the most significant adverse effect of dose intensification can be rapidly addressed [17].

In our study, we aimed to show data from our single-centre experience regarding the efficacy of salvage HDCT + ASCT in patients with relapsed/refractory EGGCT.

## 2. Materials and Methods

### 2.1. Study Design and Patient Selection

This study was a retrospective cross-sectional study. The study population consisted of male patients aged 18 years and older with recurrent/refractory EGGCTs who underwent HDCT and ASCT at the Bone Marrow Transplant Unit of Gülhane Training and Research Hospital between November 2016 and January 2023. Patients who had received at least one line of platinum-containing chemotherapy and subsequently relapsed were selected for HDCT and ASCT. Patients younger than 18 years, female patients, and patients without medical records were excluded. Clinical characteristics and follow-up data, including age, histology, metastatic site, number of lines of treatment prior to HDCT, serum alpha-fetoprotein (AFP) and beta-human chorionic gonadotropin (b-HCG) levels prior to HDCT, International Prognostic Factor Study Group (IPFSG) classification, incidence of febrile neutropenia, objective response rate (ORR), disease control rate (DCR), progression-free survival (PFS), overall survival (OS) and complete response (CR) status, and factors affecting CR were analysed.

### 2.2. High-Dose Chemotherapy Regimen and ASCT

Due to regulatory restrictions in our country, patients can undergo 1 cycle of ASCT. Therefore, all patients underwent HDCT + ASCT for consolidation after 3 cycles of TIP induction. Before ASCT, 10 mcg/kg of granulocyte-colony-stimulating factor (GCSF) was administered subcutaneously for 5 days for CD34+ stem cell collection. Stem cells were then collected. All patients received carboplatin and etoposide (CE) as an HDCT regimen. Carboplatin (700 mg/m^2^, Koçak Farma, Tekirdag, Turkey) and etoposide (750 mg/m^2^, Koçak Farma, Tekirdag, Turkey) were administered on days 1, 2, and 3. Stem cell reinfusion was performed after 2 days of rest. All patients received 500 mg of oral levofloxacin, 400 mg of oral fluconazole, and 400 mg of oral acyclovir for prophylaxis against infection. In addition, prophylactic antiemetics and oral care products were also included in the treatment regimen. Complete blood counts were performed daily until the patient achieved engraftment. Platelet engraftment was defined as a platelet count of at least 20,000/mm^3^ for three consecutive days, while neutrophil engraftment was defined as a neutrophil count of at least 2000/mm^3^. Platelet and erythrocyte suspensions were transfused to maintain levels of 20,000 platelets per mm^3^ and 8 g of erythrocytes per dL, respectively.

### 2.3. Outcomes

Platinum-refractory disease was defined as tumour progression within 4 weeks of the last cisplatin-based chemotherapy. The primary endpoint was overall survival (OS). Progression-free survival (PFS) is defined as the time from transplantation to the occurrence of disease progression, while overall survival (OS) is defined as the time from transplantation to death or last follow-up. Radiological response was evaluated by positron emission tomography/computed tomography (PET-CT) 1.5–2 months after treatment. Radiological evaluation was assessed by a trained radiologist according to the RECIST 1.1 criteria. Complete remission (CR) is defined as the absence of radiologically active residual lesions and negative blood biomarkers. Partial response (PR) is defined as a 50% reduction in the sum of the product of the longest diameters of measurable lesions or a reduction in elevated serum markers by greater than 90%. SD was defined as there being no objective tumour regression evidenced by there being no decrease in the sum of the longest diameters of any measurable lesion and no objective increase in tumour burden evidenced by no increase in the product of the longest diameters of any measurable lesion. PD was defined as an increase of more than 25% in the product of the longest diameters of any measurable lesion, the appearance of new lesions, or an increase in serum markers. ORR was calculated as the proportion of all patients achieving complete response (CR) and partial response (PR), while DCR was calculated as the proportion of all patients achieving CR, PR, and stable disease (SD). Patients who achieved CR after transplantation were followed up without treatment. Patients (platinum refractory) with PR and marker-positive SD were treated with oral etoposide, while those with progressive disease (PD) were treated with GemPOX (Gemcitabine/Paclitaxel/Oxaliplatin) regimen.

### 2.4. Statistical Analysis

Statistical analyses were performed using the SPSS version 22.0 software. The continuous independent variables were analysed using the Mann-Whitney U and Student *t*-test. Visual (histogram) and analytical (the Kolmogorov–Smirnov/Shapiro–Wilk test) methods were used to determine the distribution of variables. Normally distributed continuous variables were expressed as the mean ± standard deviation, while non-normally distributed variables were expressed as the median. The Kaplan–Meier survival function analyses and log-rank tests were used to calculate cumulative survival and treatment correlations. The categorical data were analysed for significance using Pearson’s chi-squared and Fisher’s exact tests. A *p*-value less than 0.05 was considered statistically significant. Bonferroni correction was applied to variables that were significant in the univariate analysis. Multivariate analysis was not performed because the low number of patients would affect its results.

## 3. Results

The study included 25 male patients. The median age at the time of HDCT + ASCT was 28 years (21–48). In total, 13 patients (52%) were of retroperitoneal origin, 11 patients (44%) were of mediastinal origin, and 1 patient (4%) was of brain origin. The most common histology was a mixed germ cell tumour with a rate of 52% (13 patients). According to the IPFSG classification, 12 patients (48%) were in the very high-risk group, and 5 patients (20%) were in the high-risk category. The most common sites of metastasis were the retroperitoneal lymph nodes (68%) and lungs (48%). Platinum-sensitive disease was present in 72% of patients (18 patients). Almost all patients (24 patients, 96%) had received one line of therapy prior to TIP. There were two patients (8%) with elevated AFP and four patients (16%) with elevated beta-HCG prior to HDCT + ASCT (characteristic features are shown in Table 1 and Table 2).

After HDCT + ASCT, grade 4 neutropenia, thrombocytopenia, and febrile neutropenia developed in all patients. No treatment-related deaths were observed.

In the post-transplant response evaluation, complete response (CR) was observed in seven patients (28%), partial response (PR) was observed in nine patients (36%), stable disease (SD) was observed in one patient (4%), and progressive disease (PD) was observed in eight patients (32%). The objective response rate was 64%, and the disease control rate (DCR) was 68%. The median follow-up was 25.4 months, the median progression-free survival (PFS) was 6.1 months (95% CI: 2.28–10), and the median overall survival (OS) was 12.2 months (95% CI: 6.51–17.99). The one-year CR rate was 28% and 72% (five patients) of patients who achieved CR showed disease progression within one year. Two patients were still being followed up with CR (8%). During the follow-up period, 76% (19 patients) of patients who received HDCT + ASCT died.

According to the results of the univariate analysis, the histological subtype (*p* = 0.023), presence of retroperitoneal lymph node metastasis (*p* = 0.034), AFP (*p* < 0.001) and beta-HCG elevation before HDCT (*p* = 0.039), tumour response after HDCT (*p* = 0.02), presence of CR (*p* = 0.003), objective response rate (ORR) (*p* = 0.003), and DCR (*p* = 0.004) were found to be factors influencing PFS. Although tumour location was not found to be significant for PFS, PFS was numerically better for tumours of retroperitoneal origin (9.8 months, 95% CI: 1.90–17.67). The statistically significant difference in histological subtype was due to the difference between mixed germ cell tumours and choriocarcinoma (*p* = 0.003).

In univariate analyses for OS, the histological subtype (*p* < 0.001), presence of retroperitoneal lymph node metastasis (*p* = 0.002), absence of liver metastasis (*p* = 0.02), IPFSG classification (*p* = 0.001), platinum sensitivity (*p* = 0.004), beta-HCG (*p* < 0.0001) and AFP elevation before HDCT (*p* = 0.003), tumour response after HDCT (*p* < 0.0001), presence of CR (*p* = 0.009), ORR (*p* < 0.0001), and DCR (*p* < 0.0001) were identified as factors influencing OS (Table 3).

**Figure 1:**
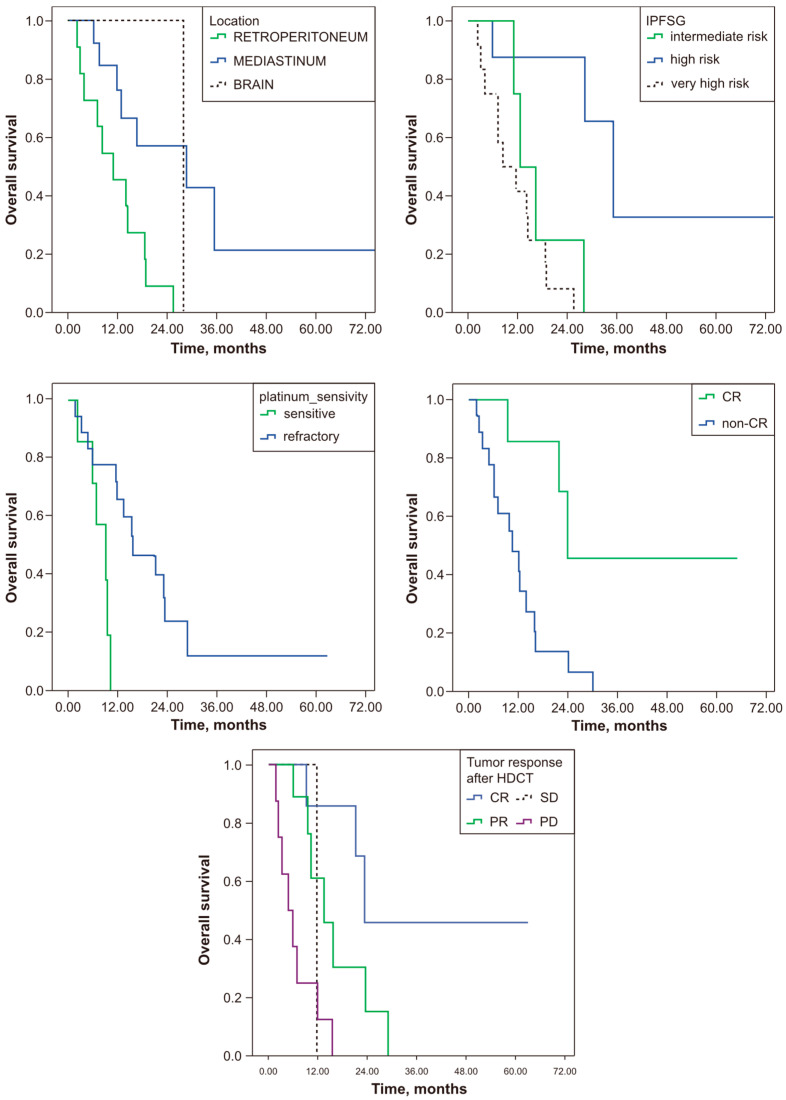
The Kaplan–Meier estimates of overall survival.

Tumour location was found to be significant for OS. Significance was found between the retroperitoneum and mediastinum, in favour of the retroperitoneum (*p* = 0.008). The significance of the histological subtype for OS was due to the difference between the yolk sac and choriocarcinoma (*p* = 0.004, in favour of the yolk sac) and mixed germ cell tumours and choriocarcinoma (*p* < 0.001, in favour of mixed). The significance of the IPFSG classification for OS was due to the difference between intermediate and high risk (*p* = 0.015, in favour of intermediate) and intermediate and very high risk (*p* = 0.001, in favour of intermediate). The significance of tumour response after HDCT for OS was due to the difference between CR and PD (*p* = 0.001, in favour of CR) and between PR and PD (*p* = 0.008, in favour of PR) (Figure 1: the Kaplan–Meier estimates of overall survival). Multivariate analysis was not performed because the small number of patients would have biased the results.

The median OS for patients with CR was 23.9 months (non-CR: 10.6 months), which was statistically significant (*p* = 0.009). When analysing factors affecting CR, none were found to predict CR (Table 4). While six of the eight patients with PD after HDCT + ASCT received GemPOx (two patients could not receive chemotherapy due to their low performance status), four platinum-resistant patients with PR + SD responses received oral etoposide. Oral etoposide was given to four platinum-sensitive patients with PR + SD responses due to elevated markers. After HDCT, patients 6, 16, 20, and 25 underwent surgery for residual tumours.

## 4. Discussion

In our study, mediastinal origin, IPFSG high-/very high-risk group, platinum-refractory disease, high AFP and beta-HCG levels prior to HDCT, and the presence of liver metastases were associated with worse OS. The median PFS was 6.1 months, and the median OS was 12.2 months at 25 months of follow-up. After approximately 2 years of follow-up, two patients (8%) were disease-free (the two patients were platinum-sensitive).

Previous studies have shown that salvage chemotherapy has worse outcomes in EGGCTs than in testicular GCTs [13,18,19]. In a study conducted at Indiana University, 12 patients with primary mediastinal non-seminomatous GCTs (PMNSGCTs) who underwent HDCT + ASCT did not have a complete response. The median survival was only 107 days. Five of the twelve patients were able to receive two cycles of HDCT + ASCT [20]. Saxman et al. reported a large series on the results of salvage chemotherapy in patients with EGGCTs. All 73 patients analysed had EGGCTs with non-seminomatous histology. In contrast to the results of salvage chemotherapy in patients with testicular germ cell tumours, only 7% of their patients achieved long-term disease-free survival. Eight patients received HDCT as a first-line salvage therapy, and twenty-eight patients received it as a third-line therapy. None of these 28 patients achieved long-term disease-free survival. Primary mediastinal location has been suggested as a negative prognostic factor [19]. In our study, mediastinal origin was also poorly prognostic in our study, and CR was achieved in seven patients (28%). The median OS was longer.

In their study at Memorial Sloan Kettering Cancer Centre (MSKCC), Feldman et al. reported on 107 patients treated with salvage HDCT with the TI-CE regimen. They observed that the presence of PMNSGCTs, third-line or later HDCT, the presence of lung metastases, beta-HCG ≥ 1000 IU/L, and more than three metastatic sites were associated with poor outcomes [21]. In a more recent study involving 364 patients treated with high-dose CE, the use of HDCT as a third-line or subsequent therapy, platinum-resistant disease, mediastinal origin, non-seminoma histology, and elevated beta-HCG at the start of HDCT were associated with worse PFS. In a series of 110 patients, Beyer et al., showed that non-seminomatous histology and mediastinal origin were associated with shorter failure-free survival (FFS) [22]. In our study, a seminoma/non-seminoma distinction could not be made because there was only one seminoma patient, and the number of metastatic sites and number of lines before HDCT had no effect on OS. The small number of patients in our study may have caused this.

The study by Pico et al. compared CDCT and HDCT in patients with relapsed/refractory GCTs. There were 31 patients diagnosed with EGGCTs in the general population. The 2-year OS of mediastinal GCTs was 22%, while the OS of retroperitoneal GCTs was 51%. The same study showed that a single course of high-dose triplet salvage chemotherapy (CarboPEC; carboplatin + etoposide + cyclophosphamide) after three cycles of conventional cisplatin-based chemotherapy was no more effective than four cycles of conventional-dose combination chemotherapy [23]. In the HDCT + ASCT study by De Giorgi et al., in 59 patients with EGGCTs, the 1- and 2-year survival rates for patients with mediastinal EGGCTs were 46% and 23%, respectively. The 1- and 2-year survival rates for patients with retroperitoneal EGGCTs were 76% and 48%, respectively. The CR rate was 43% in patients with retroperitoneal EGGCTs and 23% in patients with mediastinal EGGCTs [24]. In our study, the 1-year and 2-year OS was 59% and 30%, respectively. The CR rate of retroperitoneal EGGCTs was 30.8%, while the CR rate of mediastinal EGGCTs was 18.2%. The low survival and CR rates compared with the literature may be due to the fact that our patient population consisted of relatively high-risk patients.

A study by Richardson et al. published in May 2024 reported the results of HDCT + ASCT in 32 patients with relapsed PMNSGCTs. A total of 26 of 32 patients (81%) received two cycles of HDCT, while 6 patients (19%) received one cycle of HDCT. The 2-year PFS rate was 31%, and the 2-year OS rate was 35%. Nine patients (28%) were reported to have disease-free follow-up [25]. In a study by Adra et al., 20 patients had mediastinal origin. The 2-year PFS rate of these patients was 23%, and five of these patients (20%) were reported to be disease-free [14]. In a study by Loehrer et al., evaluating the efficacy of vinblastine, ifosfamide, and cisplatin (VelP) as a second-line therapy in patients with recurrent germ cell tumours, none of the 32 patients with nonseminomataous extragonadal tumours were disease-free compared with 100 patients with gonadal primaries [13]. Josefsen et al. reported the results of salvage therapy in 55 patients with recurrent germ cell tumours, 12 of whom had extragonadal primary tumours. Long-term disease-free survival was achieved in 3 (25%) of 12 extragonadal patients [26]. In a series of six patients in which Kumano et al. evaluated HDCT + ASCT responses in extragonadal germ cell patients, PR response was obtained in five patients and SD was observed in one patient. HDCT + ASCT was performed between one and six cycles. Five patients are still being followed up as disease-free [27]. Rosti et al. evaluated the efficacy of HDCT and ASCT in 22 extragonadal patients. CarboPEC was used as an induction regimen in the majority of patients. A median of four cycles of HDCT and ASCT were performed. CR was achieved in 17 patients. At 50 months follow-up, 15 of 17 patients were disease-free [28]. In our study, the low long-term CR rate was likely due to the high proportion of high-risk patients and the fact that one cycle of HDCT + ASCT was performed. The different induction regimens used may also have caused the responses to be different. Motzer et al. reported an overall survival rate of 20% after a median follow-up of 37 months following salvage chemotherapy. Of these 94 patients, 14 (15%) had extragonadal primary tumours, and none of these patients were alive 2 years after salvage treatment. In this study, the primary site was not a significant predictor of response to salvage chemotherapy, but there was a trend towards lower CR rates in patients with extragonadal primaries [29].

Looking at the literature for prognostic factors, the presence of non-pulmonary visceral metastasis (NPVM) was found to be a poor prognostic factor in an analysis of patients with metastatic nonseminomatous GCTs. The presence of NPVM directly places the patient at poor risk [30]. In our study, the OS of patients with liver metastases was significantly shorter.

Many studies have shown that platinum-refractory disease has poor prognostic value. In a series of 63 patients with platinum-refractory disease by Vaena et al., long-term survival was achieved in 37% of patients, and no patient with mediastinal origin achieved 2-year disease-free survival [31]. Long-term disease-free survival was achieved in 9 patients in the 32-patient series of Richardson et al. Only two of these patients had platinum-refractory disease [25]. Adra et al. included 364 patients with germ cell tumours who underwent HDCT + ASCT. While the 2-year OS rate of platinum-refractory patients was 37%, it was 80% in platinum-sensitive patients [14]. Our study supports the literature. The median OS of platinum-sensitive patients was significantly longer.

We analysed the IPFSG classification because Connolly et al. showed that the IPFSG classification is prognostic [32]. In a study by Lorch et al., the 2-year PFS rate of patients in the very low-risk group was 92%, while it was 64% in the low-risk group, 53% in the intermediate-risk group, 33% in the high-risk group, and 22% in the very high-risk group [33]. In our study, the IPFSG classification was significant for OS. There were no patients in the very low-risk and low-risk groups in our study. According to the IPFSG classification, 48% of our patients were in the very high-risk group, and 20% were in the high-risk group. This suggests that a large proportion of our patient population will have a poor prognosis. Indeed, our results confirmed this. The median OS was 30 months in the intermediate-risk group, 10.6 months in the high-risk group, and 7 months in the very high-risk group. In addition, 9 out of 12 patients with progression within 6 months after HDCT were in the high-risk or very high-risk group.

AFP and beta-HCG are important in the diagnosis, follow-up, and prognosis of GCTs [34]. Beyer et al. showed that high AFP and beta-HCG levels were associated with non-response to HDCT in a series of 110 patients (48 extragonadal patients) undergoing HDCT + ASCT [22]. Rodney et al. analysed mediastinal GCTs in a series of 635 patients and found that beta-HCG ≥ 1000 IU/L was associated with poor prognosis [35]. Our study showed that patients with AFP and beta-HCG ≥ 1000 IU/L after TIP (before HDCT) had worse survival.

In total, both CDCT and HDCT have curative potential in the salvage management of relapsed EGGCTs. Common salvage CDCT regimens include VeIP, TIP, and GIP. Although the best results reported to date have been with TIP in a favourably selected patient population, there are no randomised trials to show which regimen is superior. Salvage HDCT regimens can achieve durable remissions even in patients with unfavourable characteristics. With conflicting data from retrospective series showing better outcomes with HDCT and the IT-94 randomised trial showing no superiority of HDCT over CDCT, the optimal initial salvage approach remains unclear [36]. The TIGER trial (clinicaltrials.gov ID: NCT02375204) was designed to answer this question. It compares TIP with TI-CE and aims to definitively answer this important question. The results of this trial are yet to be obtained.

Our study has several limitations. Firstly, this was a retrospective study, and the data came from a single centre. Our patient population was small, and the proportion of high-risk patients was high. The fact that multiple cycles of HDCT + ASCT could not be applied to the high-risk patient group due to the limitations of the healthcare system is a point of criticism of our study.

Many salvage chemotherapy series for patients with metastatic germ cell tumours include patients with extragonadal primary tumours [22,23,29]. However, patients with EGGCTs are usually a small subpopulation and have rarely been studied separately [25,37]. Our study is one of the few studies to evaluate this population separately.

In summary, our results are generally poor compared with those in the literature. The main reason for this is that only one cycle of HDCT + ASCT could be performed in our high-risk patient group. A second reason is that most of our patients were at high risk according to the literature. It can be accepted that TIP induction (three cycles) followed by one cycle of HDCT + ASCT did not reflect routine salvage HDCT and was insufficient for our high-risk patient group. It is also known from the literature that patients who receive one cycle of HDCT + ASCT have worse survival rates than patients who receive multiple cycles of HDCT + ASCT [20,24,38]. Nevertheless, HDCT + ASCT has been shown to be curative in some patients.

## 5. Conclusions

Despite the small sample size, our study is one of the largest case series evaluating the outcomes of salvage HDCT in EGGCTs. Our results support that one cycle of HDCT + ASCT may not be sufficient for high-risk patients. Our study is valuable in showing that durable remission is possible in platinum-sensitive and low-risk patients. Although the CR rates in our study are low, it provides evidence that a cure can be achieved in patients with CR. In a rare disease such as an EGGCT, HDCT and ASCT may be an appropriate treatment regimen that can be used as a second line and then salvage therapy.

## Figures and Tables

**Table 1:** Patients’ characteristics.

	Age	Histology	Location	Tumour Markers at HDCT (IU/L)	IPFSG Group	Outcomes	PFS (Months)	OS (Months)	Status
1	21	Teratoma	Mediastinum	AFP: 2.75 B-HCG: 0.6	very high	PD	3	7.1	DOD
2	25	Yolk sac	Mediastinum	AFP: 429 B-HCG: 0.5	very high	PD	2.4	12.2	DOD
3	28	Mixed Germ cell	Retroperitoneum	AFP: 1.3 B-HCG: 0.7	high risk	PR	10.6	10.6	DOD
4	34	Yolk sac	Retroperitoneum	AFP: 2232 B-HCG: 0.1	very high	PD	2.4	6.1	DOD
5	22	Seminoma	Mediastinum	AFP: 3.1 B-HCG: 0.5	high risk	CR	4.1	9.4	DOD
6 *	29	Yolk sac	Mediastinum	AFP: 33 B-HCG: 1.54	very high	PR	12.5	16.1	DOD
7	26	Mixed Germ cell	Mediastinum	AFP: 4 B-HCG: 4.23	very high	PD	14.1	16	DOD
8	31	Mixed Germ cell	Retroperitoneum	AFP: 3.3 B-HCG: 0.2	intermediate	PR	10.2	10.2	PD
9	45	Mixed Germ cell	Retroperitoneum	AFP: 3.53 B-HCG: 4.33	high risk	PR	7.3	7.3	PD
10	28	Mixed Germ cell	Mediastinum	AFP: 81.5 B-HCG: 0.5	very high	CR	19	21.8	DOD
11	48	Mixed Germ cell	Retroperitoneum	AFP: 2.5 B-HCG: 8950	very high	PR	9.8	9.8	DOD
12	31	Mixed Germ cell	Mediastinum	AFP: 6900 B-HCG: 0.5	very high	PD	1.4	3.4	DOD
13	27	Yolk sac	Retroperitoneum	AFP: 3.4 B-HCG: 1.2	intermediate	CR	4.5	23.8	PD
14	25	Choriocarcinoma	Mediastinum	AFP: 1.05 B-HCG: 100,000	very high	PD	2.4	2.4	DOD
15	31	Mixed Germ cell	Retroperitoneum	AFP: 10 B-HCG: 13,800	intermediate	PD	2.6	4.9	DOD
16 *	32	Embryonal Carcinoma	Retroperitoneum	AFP: 3.54 B-HCG: 0.1	intermediate	PR	7.8	24.1	DOD
17	25	Mixed Germ cell	Retroperitoneum	AFP: 4.84 B-HCG: 2.22	intermediate	CR	25.4	25.4	NED
18	32	Yolk sac	Retroperitoneum	AFP: 115 B-HCG: 0.1	intermediate	CR	65	65	NED
19	22	Mixed Germ cell	Brain	AFP: 7 B-HCG: 241	high risk	CR	5	24	DOD
20 *	27	Mixed Germ cell	Retroperitoneum	AFP: 4 B-HCG: 0.5	intermediate	PR	4.8	30	DOD
21	25	Yolk sac	Mediastinum	AFP: 9 B-HCG: 1	very high	PR	6.1	6.1	DOD
22	25	Mixed Germ cell	Retroperitoneum	AFP: 30.7 B-HCG: 0.5	high risk	PR	3.6	14	DOD
23	34	Mixed Germ cell	Retroperitoneum	AFP: 10 B-HCG: 1.3	intermediate	CR	11	14	PD
24	43	Choriocarcinoma	Mediastinum	AFP: 2.4 B-HCG: 19,991	very high	PD	1.9	1.9	DOD
25 *	46	Teratoma	Mediastinum	AFP: 7.3 B-HCG: 0.7	very high	SD	7.2	12	DOD

AFP: alpha-fetoprotein; B-HCG: beta-human chorionic gonadotropin; CR: complete response; PR: partial response; SD: stable disease; PD: progressive disease; NED: no evidence of disease; DOD: dead of disease; IPFSG: International Prognostic Factor Study Group; IU/L: International unit/L, *: Patients undergoing residual tumour resection after HDCT.

**Table 2:** Patients’ clinical characteristics.

		n, (%)
**Number of Patients, n**		25 (100)
**Age, Median**		28 (21–48)
**Gender**	**Male**	25 (100)
Tumour Location	Retroperitoneum	13 (52)
	Mediastineum	11 (44)
	Brain	1 (4)
Histology	Seminoma	1 (4)
	Yolk Sac	6 (24)
	Embryonal Carcinoma	1 (4)
	Choriocarcinoma	2(8)
	Teratoma	2 (8)
	Mixed Germ Cell Tumour	13 (52)
Metastatic Site	Retroperitoneal Lymph Node	17 (68)
	Lung	12 (48)
	Liver	3 (12)
	Brain	2 (8)
	Bone	1 (4)
IPFSG Classification	Low	0 (0)
	Intermediate	8 (32)
	High	5 (20)
	Very High	12 (48)
Platin Sensitivity	Sensitive	18 (72)
	refractory	7 (28)
AFP Before HDCT	<1000 IU/L	23 (92)
	≥1000 IU/L	2 (8)
Beta-HCG Before HDCT	<1000 IU/L	21 (84)
	≥1000 IU/L	4 (16)
Number of Treatment Lines Before HDCT	1	24 (96)
	≥2	1 (4)
Tumour Response After HDCT	CR	7 (28)
	PR	9 (36)
	SD	1 (4)
	PD	8 (32)
ORR After HDCT	Present	16 (64)
	Absent	9 (36)
DCR	Present	17 (68)
	Absent	8 (32)
CR STATUS	CR	7 (28)
	Non-CR	18 (72)
STATUS	Alive	6 (24)
	Dead	19 (76)

IPFSG: International Prognostic Factor Study Group; AFP: alpha-fetoprotein; Beta-HCG: beta-human chorionic gonadotropin; HDCT: high-dose chemotherapy; ORR: objective response rate; DCR: disease control rate; CR: complete response; IU/L: international unit/litre.

**Table 3:** Analysis of patients for progression-free survival and overall survival according to clinicopathological factors.

	Median PFS (Months) (95% CI)		*p*	OS (Months) (95% CI)	*p*
PFS, OS (Months)	6.1 (2.28–10.0)			12.25 (6.51–17.99)	
Tumour Location					
Retroperitoneum	9.79 (1.90–17.67)		0.35	24.14 (12.72–42.83)	0.013
Mediastineum	4.41 (0.13–8.14)			9.42 (6.06–13.64)	
Brain	4.92 (4.92–4.92)			23.95 (23.95–23.95)	
Histology					
Seminoma	4.14 (4.14–4.14)		0.023	9.42 (9.42–9.42)	<0.0001
Yolk Sac	4.46 (0.12–8.92)			12.25 (7.55–49.27)	
Embryonal Carcinoma	7.75 (7.75–7.75)			24.14 (24.14–24.14)	
Choriocarcinoma	1.87 (1.87–1.87)			1.87 (1.87–1.87)	
Teratoma	2.95 (2.95–2.95)			7.09 (4.73–14.39)	
Mixed Germ Cell Tumour	10.61 (2.12–19.09)			15.90 (5.34–26.46)	
Metastatic Site					
Retroperitoneal Lymph Node	Present	9.79 (3.02–16.55)	0.034	15.90 (3.17–28.63)	0.027
	Absent	2.43 (0.0–5.25)		6.14 (0.0–18.16)	
Lung	Present	4.46 (1.06–7.87)	0.36	9.42 (4.00–14.85)	0.059
	Absent	7.19 (3.97–10.41)		23.95 (5.87–42.03)	
Liver	Present	2.95 (2.43–3.48)	0.23	7.09 (3.57–10.61)	0.023
	Absent	6.14 (2.87–9.41)		15.90 (10.26–27.73)	
Brain	Present	4.92 (4.92–4.92)	0.86	10.61 (4.20–30.35)	0.91
	Absent	6.14 (1.87–10.41)		13.89 (8.67–19.12)	
Bone	Present	2.43 (2.43–2.43)	0.08	12.25 (12.25–12.25)	0.68
	Absent	6.14 (2.72–9.56)		13.89 (10.77–26.29)	
IPFSG Classification					
Low	0 (0–0)		0.23	0 (0–0)	0.001
Intermediate	7.75 (0.37–15.13)			29.69 (21.12–38.8)	
High	4.92 (3.23–6.62)			10.61 (6.23–14.99)	
Very High	2.95 (0.0–9.25)			7.09 (0.90–13.28)	
Platin Sensitivity					
Sensitive	6.14 (1.43–10.85)		0.26	16.09 (6.51–25.68)	0.004
Refractory	4.14 (1.10–7.17)			9.42 (4.64–14.21)	
AFP Before HDCT					
<1000 IU/L	7.19 (2.91–11.47)		<0.0001	13.9 (8.51–19.27)	0.003
≥1000 IU/L	1.38 (1.38–1.38)			3.35 (3.35–3.35)	
Beta-HCG Before HDCT					
<1000 IU/L	7.19 (3.12–11.26)		0.039	15.9(10.90–20.90)	<0.0001
≥1000 IU/L	2.43 (1.69–3.17)			2.43 (0–5.39)	
Number of Lines Before HDCT					
1	4.92 (1.65–8.20)		0.9	12.25 (7.49–17.01)	0.61
≥2	7.75 (7.75–7.75)			24.18 (24.18–24.18)	
Tumour Response After HDCT					
CR	11.03 (0.0–26.72)		0.012	23.95 (23.95–23.95)	<0.0001
PR	9.79 (5.13–14.45)			13.89 (7.22–20.56)	
SD	7.19 (7.19–7.19)			12.02 (12.02–12.02)	
PD	2.43 (2.34–2.51)			4.89 (1.11–8.67)	
CR Status					
CR	11.03 (0.0–26.72)		0.049	23.95 (23.95–23.95)	0.009
Non-CR	3.54 (0–10.22)			10.61 (6.72–14.49)	
DCR					
Present	9.79 (5.79–13.78)		0.001	21.78 (11.11–32.45)	<0.0001
Absent	2.43 (2.34–2.52)			4.89 (1.11–8.67)	
ORR After HDCT					
Present	9.79 (5.03–14.55)		0.003	21.78 (10.06–33.50)	<0.0001
Absent	2.43 (2.33–2.52)			6.07 (2.62–9.53)	
Median Follow-up Time (Months)	25.42				
12 Months PFS%	25				
24 Months PFS%	10				
12 Months OS%	59				
24 Months OS%	30				

PFS: progression-free survival; OS: overall survival; IPFSG: International Prognostic Factor Study Group; AFP: alpha-fetoprotein; Beta-HCG: beta-human chorionic gonadotropin; HDCT: high-dose chemotherapy; ORR: objective response rate; DCR: disease control rate; CR: complete response; PR: partial response; SD: stable disease; PD: progressive disease; IU/L: international unit/litre.

**Table 4:** Factors influencing the probability of complete response.

		CR	Non-CR	*p*
		n, %	n, %	
Location	Retroperitoneum	4 (16)	9 (36)	0.2
	Mediastineum	2 (8)	9 (36)	
	Brain	1 (4)	0 (0)	
Histology	Seminoma	1 (4)	0 (0)	0.46
	Yolk Sac	2 (8)	4 (16)	
	Embryonal Carcinoma	0 (0)	1 (4)	
	Choriocarcinoma	0 (0)	2 (8)	
	Teratoma	0 (0)	2 (8)	
	Mixed Germ Cell Tumour	4 (16)	9 (36)	
Number of lines before HDCT	1	7 (28)	17 (68)	0.72
	≥2	0 (0)	1 (4)	
IPFSG Classification	Low	0 (0)	0 (0)	0.1
	Intermediate	4 (16)	4 (16)	
	High	2 (8)	3 (12)	
	Very High	1 (4)	11 (44)	
AFP Before HDCT	<1000 IU/L	7 (28)	16 (64)	0.51
	≥1000 IU/L	0 (0)	2 (8)	
Beta-HCG Before HDCT	<1000 IU/L	7 (28)	14 (68)	0.29
	≥1000 IU/L	0 (0)	4 (16)	
Metastatic site	Retroperitoneal Lymph Node	6 (24)	11 (44)	0.24
	Lung	3 (12)	9 (36)	0.55
	Liver	0 (0)	3 (12)	0.35
	Brain	1 (4)	1 (4)	0.49
	Bone	0 (0)	1 (4)	0.52
Platinum Sensitivity	Sensitive	6 (24)	12 (48)	0.33
	Refractory	1 (4)	6 (24)	

IPFSG: International Prognostic Factor Study Group; AFP: alpha-fetoprotein; Beta-HCG: beta-human chorionic gonadotropin; HDCT: high-dose chemotherapy; ORR: objective response rate; CR: complete response; PR: partial response; SD: stable disease; PD: progressive disease.

## Data Availability

The raw data supporting the conclusions of this study will be made available by the authors upon request.
